# Molecular Mechanisms Underlying the Regulation of Biofilm Formation and Swimming Motility by FleS/FleR in *Pseudomonas aeruginosa*

**DOI:** 10.3389/fmicb.2021.707711

**Published:** 2021-07-21

**Authors:** Tian Zhou, Jiahui Huang, Zhiqing Liu, Zeling Xu, Lian-hui Zhang

**Affiliations:** ^1^Guangdong Laboratory for Lingnan Modern Agriculture, South China Agricultural University, Guangzhou, China; ^2^Guangdong Province Key Laboratory of Microbial Signals and Disease Control, Integrative Microbiology Research Center, South China Agricultural University, Guangzhou, China

**Keywords:** *Pseudomonas aeruginosa*, two-component system, FleS/FleR, biofilm, swimming motility

## Abstract

*Pseudomonas aeruginosa*, a major cause of nosocomial infection, can survive under diverse environmental conditions. Its great adaptive ability is dependent on its multiple signaling systems such as the two-component system (TCS). A TCS FleS/FleR has been previously identified to positively regulate a variety of virulence-related traits in *P. aeruginosa* PAO1 including motility and biofilm formation which are involved in the acute and chronic infections, respectively. However, the molecular mechanisms underlying these regulations are still unclear. In this study, we first analyzed the regulatory roles of each domains in FleS/FleR and characterized key residues in the FleS-HisKA, FleR-REC and FleR-AAA domains that are essential for the signaling. Next, we revealed that FleS/FleR regulates biofilm formation in a c-di-GMP and FleQ dependent manner. Lastly, we demonstrated that FleR can regulate flagellum biosynthesis independently without FleS, which explains the discrepant regulation of swimming motility by FleS and FleR.

## Introduction

*Pseudomonas aeruginosa* is an opportunistic Gram-negative pathogen that causes a variety of acute and chronic infections in humans (Driscoll et al., 2007; Coggan and Wolfgang, 2012). It is the leading cause of morbidity and mortality of patients with compromised immune systems or patients suffering from cystic fibrosis (Bodey et al., 1983; Richards et al., 2000; Ahator and Zhang, 2019). *P. aeruginosa* has an unusually large genome size of 6.4 Mbp with nearly 6,000 genes including a majority of them encoding proteins associated with virulence factors, secretion systems, motility, efflux pumps, chemotaxis and biofilm formation, conferring to the pathogen great ability to infect and adapt to diverse host habitats (Silby et al., 2011; Cao et al., 2017). For example, it is highly mobile and armed with full virulence factors to establish colonization at the early acute infection then gradually shifts to an adapted situation with increased persistence and biofilm formation if not successfully eradicated by the human immune system (Sousa and Pereira, 2014). In addition, this pathogen can also survive and thrive in diverse terrestrial and aquatic niches with its unique ability in sensing and responding to environmental changes (Rahme et al., 1995).

The great capacity of *P. aeruginosa* responding to diverse external stimuli is known to be achieved by its sophisticated signal transduction networks. Among them, two-component system (TCS) is one of the major signaling systems that is employed to control gene expression profiles in response to the changing environments (Hoch and Silhavy, 1995; Rodrigue et al., 2000). TCS is typically composed of a transmembrane sensor histidine kinase (HK) and its corresponding cytoplasmic response regulator (RR) (Stover et al., 2000). Usually, the HisKA domain in the HK transduces the signal from specific stimulus by autophosphorylation at a conserved histidine residue using an adenosine triphosphate (ATP) as the substrate. Subsequently, the phosphoryl group at the histidine residue is transferred to a conserved aspartate residue in the REC domain which is usually located at the N-terminus of the cognate RR (Stover et al., 2000). The phosphorylated RR then activates or represses the expression of downstream genes or modulates cellular behaviors via its output domain by interaction with target gene promoters (Hoch and Silhavy, 1995; Stover et al., 2000). There are in total 64 HKs and 73 RRs have been identified in *P. aeruginosa* PAO1 (Rodrigue et al., 2000). They are involved in the perception of environmentally or physiologically related signals and contribute significantly to the virulence and persistence of this pathogen (Kay et al., 2006; Francis et al., 2017).

The TCS FleS/FleR was initially identified in *P. aeruginosa* PAK which is involved in its motility and adhesion to mucin and was further found to modulate swarming motility in PA14 (Ritchings et al., 1995; Kollaran et al., 2019). It was implicated that FleS/FleR modulates motility by controlling the expression of flagellum biosynthetic genes (Dasgupta et al., 2003). Despite FleS/FleR was identified two decades ago, its physiologically relevant stimuli and regulatory pathways are still elusive. Previous studies revealed that the activation of FleS/FleR requires another TCS PilS/PilR, an alternate sigma factor RpoN (σ54) and a transcriptional regulator FleQ (Arora et al., 1997; Kilmury and Burrows, 2018). Among these activators, FleQ is expressed from the locus immediately upstream of the *fleSR* operon and is known to control the transcription of FleS/FleR in a c-di-GMP dependent manner (Jimenez-Fernandez et al., 2016). In addition to the undetermined stimuli and regulation, whether this TCS exerts additional physiological functions is also largely unknown.

Previous studies reported that FleS/FleR is involved in the regulation of swimming motility and biofilm formation (Zhou et al., 2021), suggesting that this TCS might be important to switch the acute and chronic infections in *P. aeruginosa*. However, their underlying regulatory mechanisms are not clear. Distinct from canonical TCSs, some unique features were identified in FleS and FleR. For instance, FleS lacks a transmembrane domain and FleR is equipped with an additional AAA domain. Given that the special structures of this TCS, in this study, we first dissected the domain structures of FleS and FleR and attempted to investigate the molecular mechanisms underlying their regulation of biofilm formation and swimming motility. Although both phenotypes are positively regulated by FleS/FleR, we found that they are interestingly regulated in different patterns. Both FleS and FleR regulate biofilm formation which is consistent with our understanding that they function as a signaling pair. However, only FleR plays the essential role in regulating swimming motility while FleS exhibits moderate impact on swimming in PAO1.

## Materials and Methods

### Bacterial Strains and Growth Conditions

*P. aeruginosa* strains and other bacterial strains used in this study are listed in the Supplementary Table 1. *P. aeruginosa* and *E. coli* strains were routinely cultured at 37°C in lysogeny broth (LB). Antibiotics were added when necessary in the following concentrations: gentamicin, 50 μg/ml for *P. aeruginosa* and *E. coli*; ampicillin, 100 μg/ml for *E. coli*; kanamycin, 50 μg/ml for *E. coli*. Cell density was determined by measuring optical density at the wavelength of 600 nm.

### Construction of PAO1 Mutants and Complementation

The plasmids and primers used in this study were listed in Supplementary Table 2. In-frame gene deletion in *P. aeruginosa* PAO1 followed a previously published method (An et al., 2010). Briefly, the upstream and downstream homologous arms of the target gene were amplified by PCR using Pfu DNA polymerase (Vazyme, China) and ligated into pk18mobsacB which was pre-digested with *Bam*HI and *Hin*dIII, generating the plasmids for target gene deletion. The resultant plasmid was transformed into PAO1 by tri-parental mating with the help of another plasmid pRK2013. Desired gene deletion mutants were screened on the LB agar plates supplemented with 10% sucrose. The mutants were confirmed by PCR and DNA sequencing.

For complementation, the open reading frames (ORF) or different domain regions of target genes were amplified and cloned into the plasmid pBBR1-MCS5. Point mutations in the complemented genes were constructed using the QuikChange site-directed mutagenesis system (Agilent, United States). All the constructs were transformed into PAO1 wild type or its mutants using the helper plasmid pRK2013 by triparental mating. The success of plasmid delivery into the PAO1 strains was verified by PCR. Stable expression of the FleS and FleR variants constructed in this study was confirmed by western blot (Supplementary Figure 1).

### Western Blot Analysis

Western blot was performed following the procedures published by An et al. (An et al., 2010). Briefly, bacterial pellet was collected from 10 ml cell culture by centrifugation. Total proteins were extracted using EZLys^TM^ bacterial protein extraction reagent (BioVision, United States) with sonification. For each sample, proteins were separated in an 8% SDS-PAGE gel and then transferred into a PVDF membrane (Millipore, United States). The membrane was blocked using 5% non-fat milk in PBST (PBS supplemented with 1% Tween-20) followed by immunoblotting using anti-His antibody (Abbkine, United States) and HRP-conjugated IgG (Abbkine, United States). Finally, proteins on the membrane were detected using a cooled CCD camera (Tanon, China).

### Biofilm Formation Assay

Biofilm formation was assayed in 96-well polypropylene microtiter dishes according to the previously described method (An et al., 2010). The overnight bacterial culture was diluted to OD_600__nm_ of 0.002 using fresh LB medium. 200 μl diluted culture was transferred into 96-well polypropylene microtiter dishes, and then incubated at 37°C for 20 h. The cell densities of bacterial cultures were determined by a microplate reader (BioTek, United States). The bacterial cells were carefully removed and washed three times with sterile water. The biofilm cells were stained with 200 μl 0.1% crystal violet for 15 min at room temperature. After washing three times, the plates were air dried at room temperature. For quantification, the bound crystal violet was resuspended in 200 μl of 95% ethanol and measured at 570 nm with a microplate reader (BioTek, United States).

### Swimming Assay

For swimming motility, the agar plates were prepared to contain 10 g/L Tryptone, 5 g/L Yeast extract and 0.25 g/L agar. Swimming motility was assayed following a previously described method (Rashid and Kornberg, 2000). Briefly, 15 ml agar medium was poured in to a 90-mm petri dish and then the prepared plates were wrapped to prevent dehydration. 1 μl overnight culture were spotted into the center of the agar plate. The plates were incubated at 37°C for 12 h.

### RNA Extraction and Quantitative Real-Time PCR

PAO1 and its derivatives were diluted from the overnight cultures and subcultured in LB medium until the OD_600__nm_ reached 1.5. Total RNA was isolated using the SV Total RNA separation and purification system (Promega, United States) according to the manufacturer’s instructions. The purity and integrity of RNA after genomic DNA digestion (Invitrogen, United States) were assessed by UV spectrophotometry and agarose gel electrophoresis. The first-strand cDNA was reversely transcribed from the isolated total RNA by using the HiScript III 1st Strand cDNA Synthesis Kit (with gDNase) (Vazyme, China). Real-time PCR was performed using the ChamQ Universal SYBR qPCR master mix (Vazyme, China) on the ABI QuantStudioTM6 Flex system according to the manufacturer’s instructions. The real-time PCR primers were listed in Supplementary Table 2. The 50S ribosomal protein gene *rplU* was selected as the internal control. The relative gene expression level was calculated by using 2^–ΔΔCT^ method (Kuchma et al., 2007). The experiment was performed in triplicates.

### Transmission Electron Microscopy (TEM)

PAO1 and its mutants were grown in LB medium overnight at 37°C with shaking. Cells were diluted and stained with 2% aqueous solution of phosphotungstic acid (pH 7.4) for 20 min. Then, the cells were fixed for 5 min in Parlodion (Mallinckrodt, Inc., St. Louis, MO, United States) carbon-coated grid (300 mesh). Samples were examined with a Talos F200S (Thermo Fisher) transmission electron microscope operating at 200 kV. The experiment was repeated three times.

### Statistical Analysis

Experimental data were analyzed by one-way analysis of variance (ANOVA) and means were compared by Bonferroni’s multiple comparison test using Graphpad Prism software (version 8). Experiments were arranged as completely randomized design and differences at *P* < 0.05 were considered as statistically significant. ^∗^, ^∗∗^, and ^∗∗∗^ indicated *P* < 0.05, *P* < 0.01, and *P* < 0.001, respectively.

## Results

### Functional Domain Analysis of the TCS FleS and FleR

**FIGURE 1:**
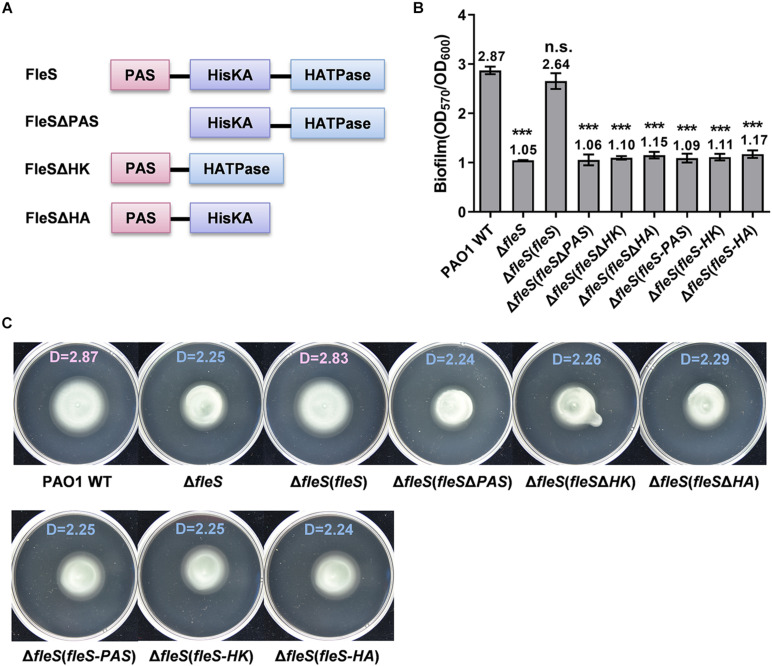
PAS, HisKA, and HATPase domains in FleS are all essential for its signaling to regulate biofilm formation and swimming motility. **(A)** A schematic diagram showing the domain structures of FleS and its variants. Domain structures were predicted using the SMART program (http://smart.embl-heidelberg.de/). **(B)** Biofilm formation of the PAO1 wild type (WT), the ?*fleS* mutant and the ?*fleS* mutant with ectopic expression of FleS, FleSΔPAS, FleSΔHK, FleSΔHA as well as the single FleS domains FleS-PAS, FleS-HK, and FleS-HA. The result is shown as the mean of five replicates per strain and error bar indicates the standard error of mean (SEM). ***P < 0.001, n.s., no significance (Student’s *t*-test). **(C)** Swimming motility of the PAO1 wild type, the Δ*fleS* mutant and the Δ*fleS* mutant with ectopic expression of FleS, FleSΔPAS, FleSΔHK, FleSΔHA, FleS-PAS, FleS-HK, and FleS-HA. Representative images and the average diameter (D) of the swimming migration zone of each strain are shown. Each experiment was performed in triplicate.

In addition to control swarming motility (Kollaran et al., 2019), our recent study has demonstrated that the TCS FleS/FleR is also involved in the regulation of biofilm formation and swimming motility in *P. aeruginosa* PAO1 (Zhou et al., 2021). To understand the signal transduction mechanism of this TCS, we firstly conducted a comprehensive functional dissection of each domain in the sensor histidine kinase FleS and the response regulator FleR. Unlike typical histidine kinases which contain a transmembrane domain, FleS only consists of three domains namely PAS, HisKA (HK), and HATPase (HA) (Figure 1A). To determine the involvement of these individual domains in biofilm formation and swimming motility, we constructed three FleS variants with the lack of each domain in the protein, i.e., FleSΔPAS, FleSΔHK, FleSΔHA (Figure 1A), and applied these variants to perform complementation assays in the PAO1 Δ*fleS* mutant. First, biofilm formation assay showed that only ectopic expression of the full-length wild-type FleS but not its variants could restore biofilm formation of the Δ*fleS* mutant to the wild-type level (Figure 1B). Similarly, swimming motility of the Δ*fleS* mutant was only restored by the wild-type FleS rather than any other variants (Figure 1C). These results indicated that all the three domains in the HK FleS are essential in regulating biofilm formation and swimming motility in PAO1.

**FIGURE 2:**
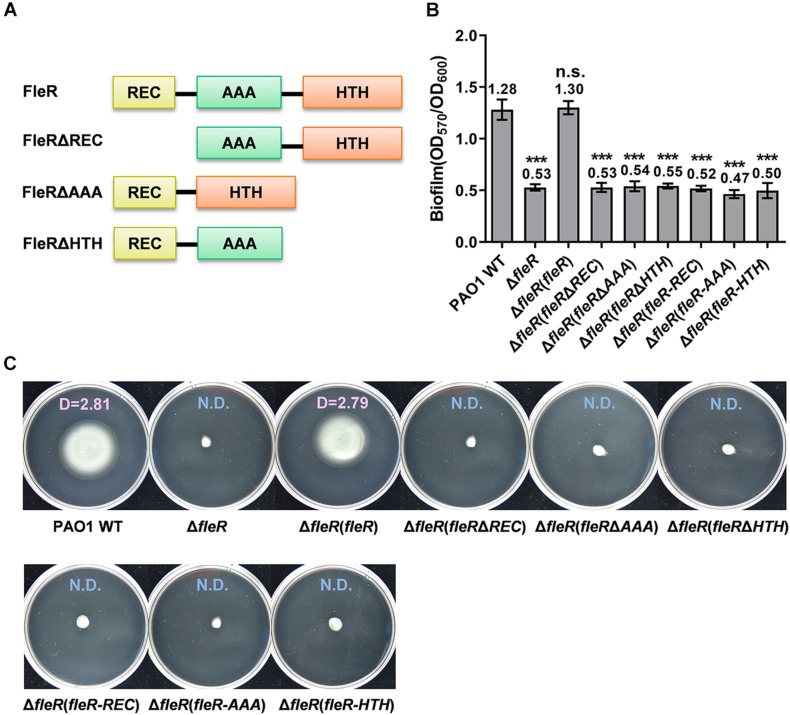
REC, AAA, and HTH domains in FleR are all essential to control biofilm formation and swimming motility. **(A)** A schematic diagram showing the domain structures of FleR and its variants. **(B)** Biofilm formation of the PAO1 wild type (WT), the Δ*fleR* mutant and the Δ*fleR* mutant with ectopic expression of FleR, FleRΔREC, FleRΔAAA, FleRΔHTH as well as the single FleR domains FleR-REC, FleR-AAA, and FleR-HTH. The result is shown as the mean of five replicates per strain and error bar indicates the standard error of mean (SEM). ***P < 0.001, n.s., no significance (Student’s *t*-test). **(C)** Swimming motility of the PAO1 wild type, the Δ*fleR* mutant and the Δ*fleR* mutant with ectopic expression of FleR, FleRΔREC, FleRΔAAA, FleRΔHTH, FleR-REC, FleR-AAA, and FleR-HTH. Representative images and the average diameter (D) of the swimming migration zone of each strain are shown. N.D., not detected. Each experiment was performed in triplicate.

Distinct from the ubiquitous RRs which contain REC and HTH domains, FleR is predicted to contain an additional domain AAA (Figure 2A). Similarly, to investigate the role of three domains in FleR, we generated three FleR variants for complementation analysis (Figure 2A). Compared to the ectopic expression of the wild-type FleR which fully restored biofilm formation and swimming motility of PAO1 Δ*fleR*, it was shown that ectopic expression of the FleR variants FleRΔREC, FleRΔAAA, FleRΔHTH in PAO1 Δ*fleR* had no effect on the recovery of its biofilm formation and swimming motility (Figures 2B,C), indicating that all the three domains are required for the FleR activity. Combined, these results demonstrated that all the domains in the TCS FleS/FleR are indispensable for its signal transduction and phenotypic regulation.

### The H191A Replacement in the HisKA Domain of FleS Abolished FleS Signaling

**FIGURE 3:**
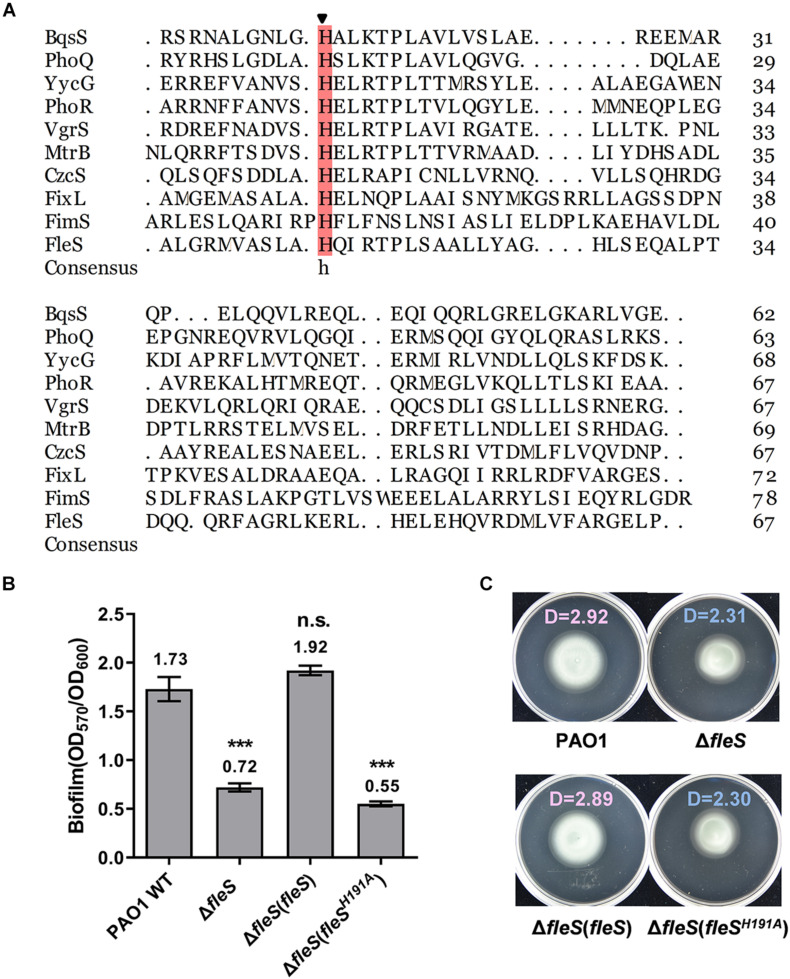
The histidine at the position of 191 in FleS is conserved with other histidine kinase and important in FleS signaling. **(A)** Sequence alignment of the HisKA domain of FleS with nine canonical HisKA domains from YycG (WP_023482385.1), PhoR (WP_003121316.1), FixL (WP_011119443.1), VgrS (WP_011038221.1), MtrB (WP_003917137.1), CzcS (WP_016253371.1), FimS (WP_003096404.1), PhoQ (WP_003082438.1), BqsS (WP_003090493.1). The arrow indicates the conserved histidine residue that is involved in phosphoryl group transfer. **(B,C)** The importance of the FleS H191 in regulating biofilm formation **(B)** and swimming motility **(C)** was examined by ectopic expression of FleS^H191A^ in the Δ*fleS* strain. ***P < 0.001, n.s., no significance (Student’s *t-*test). Representative images and the average diameter (D) of the swimming migration zone of each strain are shown. Each experiment was performed in triplicate.

PAS domains are known as versatile sensors to detect chemical and physical stimuli in signal transduction proteins (Moglich et al., 2009). Although the physiologically relevant signals of FleS-PAS are still not identified, it is conventionally known that signal perception by the PAS domain triggers downstream autophosphorylation at a conserved histidine residue in the HisKA domain by using an ATP (Tiwari et al., 2017). To explore the key histidine residue of FleS involved in signal transduction, we firstly conducted sequence alignment of its HisKA domain with nine canonical HisKA domains in PAO1 and a conserved histidine residue at the position 191 was revealed (Figure 3A). To verify whether this residue (H191) is the key histidine residue for signal transduction, we substituted the histidine residue at 191 with alanine (FleS^H191A^) and examined biofilm formation and swimming motility of PAO1 Δ*fleS* with the ectopic expression of FleS^H191A^. As expected, FleS with H191A substitution was unable to restore the biofilm formation and swimming motility of PAO1 Δ*fleS* any longer (Figures 3B,C), confirming the crucial role of H191 in the FleS signaling.

### Aspartate Residues at Positions 10, 11, 53, 60, and 99 Are Essential for the Activity of FleR

**FIGURE 4:**
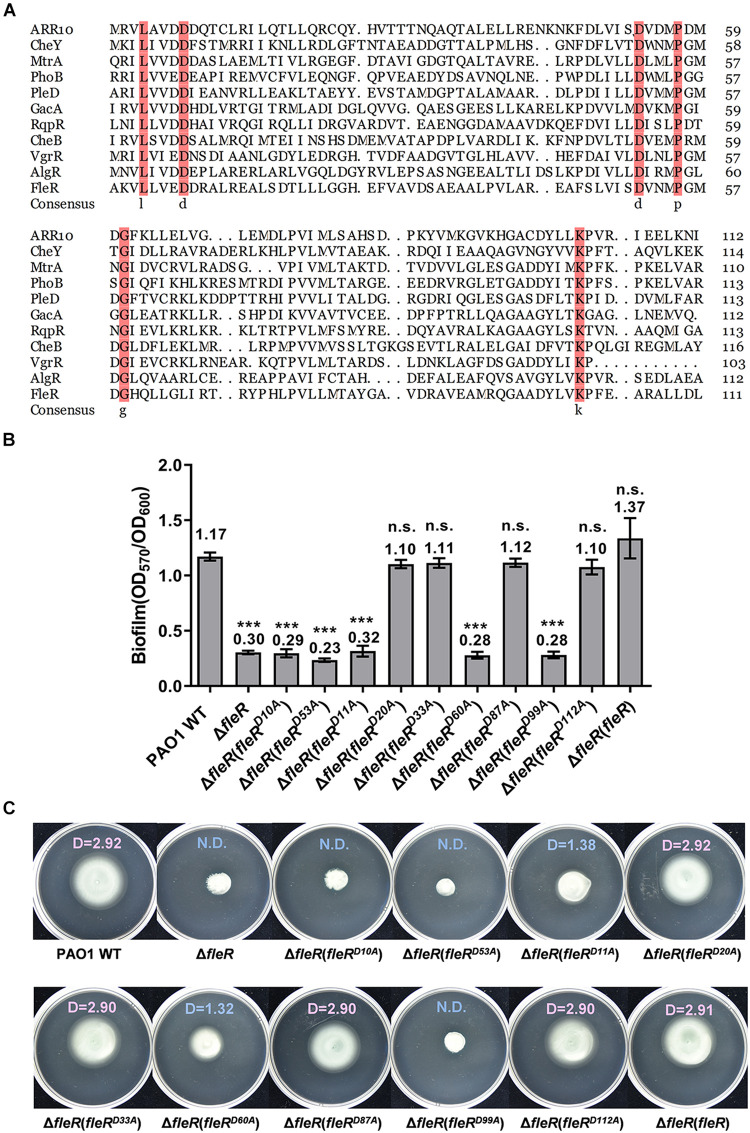
Aspartate residues at positions of 10, 11, 53, 60, 99 in the REC domain of FleR are essential for the FleR activity. **(A)** Sequence alignment of the REC domain of FleR with 10 canonical REC domain sequences from ARR10 (NP_194920.1), CheB (WP_001350517.1), NreC (CPM47396.1), MtrA (AAB07804.1), PhoB (WP_000113933.1), PleD (WP_004620047.1), VgrR (WP_003490678.1), GacA (AAA68948.1), AlgR (AAA88427.1), and RqpR (CDN61654.1). The arrows indicated the potential aspartate residue to receive phosphoryl group. **(B,C)** The role of all the aspartate residues in FleR REC in controlling biofilm formation **(B)** and swimming motility **(C)** was examined by ectopic expression of FleR containing each D to A substitution in the Δ*fleR* strain. ***P < 0.001, n.s., no significance (Student’s *t-*test). Representative images and the average diameter (D) of the swimming migration zone of each strain are shown. N.D., not detected. Each experiment was performed in triplicate.

Signaling within the TCS is achieved by the phosphoryl group which is transferred from the conserved histidine residue of the HisKA domain in the HK to the aspartate residue of the REC domain in the RR (Stover et al., 2000). To identify the aspartate residue that potentially serves as the receiver for the transferred phosphoryl group, amino acid sequence of FleR-REC was first aligned with 10 canonical REC domains for the prediction. Alignment result identified two conserved aspartate residues at position 10 (D10) and 53 (D53) (Figure 4A). To confirm the role of these two conserved aspartate residues, we first introduced the substitutions of D10A and D53A into the FleR protein, respectively, generating FleR^D10A^ and FleR^D53A^. Compared with the ectopic expression of the wild-type FleR which fully restored biofilm formation and swimming motility of PAO1 Δ*fleR*, expression of either FleR^D10A^ or FleR^D53A^ still showed the same phenotypes as the Δ*fleR* mutant (Figures 4B,C). These results indicated that more than one aspartate residues contribute to the activity of FleR. Therefore, we were interested to examine if other aspartate residues are also required for the signaling. In addition to D10 and D53, seven additional aspartate residues were found in the REC domain of FleR, i.e., D11, D20, D33, D60, D87, D99, D112 (Supplementary Figure 2). Alanine substitution was constructed at each of the seven aspartate residues in FleR and their capabilities to enhance the biofilm formation and swimming motility of PAO1 Δ*fleR* were examined, respectively. Introduction of the FleR variants with alanine substitutions at D20, D33, D87, and D112 exhibited the same results as the introduction of the wild-type FleR which enabled the recovery of biofilm formation and swimming motility of the Δ*fleR* mutant to the level as the PAO1 wild type (Figures 4B,C). However, the other three FleR variants with substitutions at D11, D60, D99 failed to restore the biofilm formation and swimming motility of the Δ*fleR* mutant (Figures 4B,C). Therefore, these results indicated that in total five aspartate residues at positions 10, 11, 53, 60 and 99 are essential for the activity of FleR. Since the aspartate residues for phosphorylation in PhoB, VgrR and AlgR were identified at positions corresponding to the D53 in FleR (Whitchurch et al., 2002; Wang et al., 2016; Meng et al., 2020), it is of high possibility that D53 serves as the receiver for the phosphoryl group in FleR.

### The Residues K164, D229&E230, T208 of the AAA Domain Are Essential for the Activity of FleR

FleR was predicted as a σ^54^-dependent transcriptional activator owing to the presence of a σ^54^-interaction region in the AAA domain (Studholme and Dixon, 2003; Seraphim and Houry, 2020). We next sought to characterize this AAA domain in FleR because AAA domain is relatively uncommon in the TCS response regulators. AAA domain is recognized by the Walker A and Walker B motifs which are involved in nucleotide binding and hydrolysis, respectively (Bush and Dixon, 2012; Seraphim and Houry, 2020). The Walker A motif is generally composed of the amino acid residues GXXXXGK[T/S] (X represents any amino acid) involved in ATP binding and proper positioning of the triphosphate group of the NTP (Seraphim and Houry, 2020). The Walker B motif is formed by hhhhDD/E residues (h represents a hydrophobic amino acid). In the case of PspF from *E. coli*, the residue K42 in the Walker A motif and the residues D107&E108 in the Walker B motif were found as the key residues to support the activity of ATP hydrolysis (Schumacher et al., 2004; Joly et al., 2007).

**FIGURE 5:**
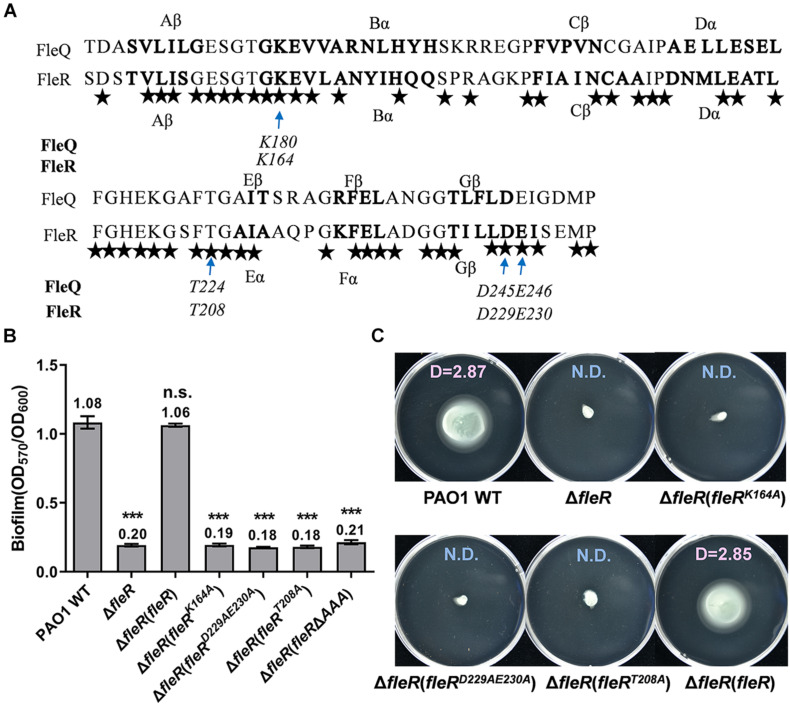
Analysis of the conserved amino acids in the AAA domain of FleR. **(A)**. The AAA domain of FleR displays a similar structural fold and shares the conserved key residues with the AAA domain of FleQ (WP_003086448.1). The α helix and β sheet of FleR and FleQ shown in bold were predicted using PredictProtein (http://cubic.bioc.columbia.edu/). The arrows indicate the conserved amino acid residues which are the key residues in the Walker A, Walker B and σ^54^-binding motifs in FleQ. All the identical residues in FleR and FleQ are indicated by stars. **(B,C)** The role of K164, D229&E230, T208 in FleR-AAA in controlling biofilm formation **(B)** and swimming motility **(C)** was examined by ectopic expression of FleR containing K164A, D229A&E230A, T208A in the Δ*fleR* strain, respectively. ***P < 0.001, n.s., no significance (Student’s *t-*test). Representative images and the average diameter (D) of the swimming migration zone of each strain are shown. N.D., not detected. Each experiment was performed in triplicate.

As shown in Supplementary Figure 3, the FleR-AAA domain shared 59% identity with the AAA domain of FleQ which is a well-studied σ^54^-dependent master regulator in *P. aeruginosa* (Arora et al., 1997; Dasgupta et al., 2003). Furthermore, the AAA domains of FleR and FleQ shared high similarity in the secondary structure fold and four conserved residues were identified, namely K164, D229&E230, T208 in FleR corresponding to the K180, D245&E246, T224 in FleQ, respectively (Figure 5A). Since it has already known that K180, D245&E246, T224 are the key residues of the Walker A motif, the Walker B motif, and the σ^54^-binding domain, respectively, in FleQ-AAA to fulfill its regulatory function (Baraquet and Harwood, 2013), we moved to investigate whether those conserved amino acids identified in FleR are also essential for its activity. Alanine substitutions at those positions were introduced into the FleR protein which was employed for the complementary assay. Single substitutions at K164 (FleR^K164A^), T208 (FleR^T208A^) and simultaneous double substitutions at D229 and E230 (FleR^D229AE230A^) were constructed. As shown in Figures 5B,C, ectopic expression of each FleR variants in PAO1 Δ*fleR* failed to restore its abilities of biofilm formation and swimming motility. The results confirmed that residues K164, D229&E230 and T208 are conserved key residues of the Walker A motif, the Walker B motif, and the σ^54^-binding region in FleR. These results also suggested that FleR functions not only as a TCS response regulator but also as a probable σ^54^-dependent transcriptional activator.

### FleS/FleR Regulates Biofilm Formation in the c-di-GMP and FleQ Dependent Manner

**FIGURE 6:**
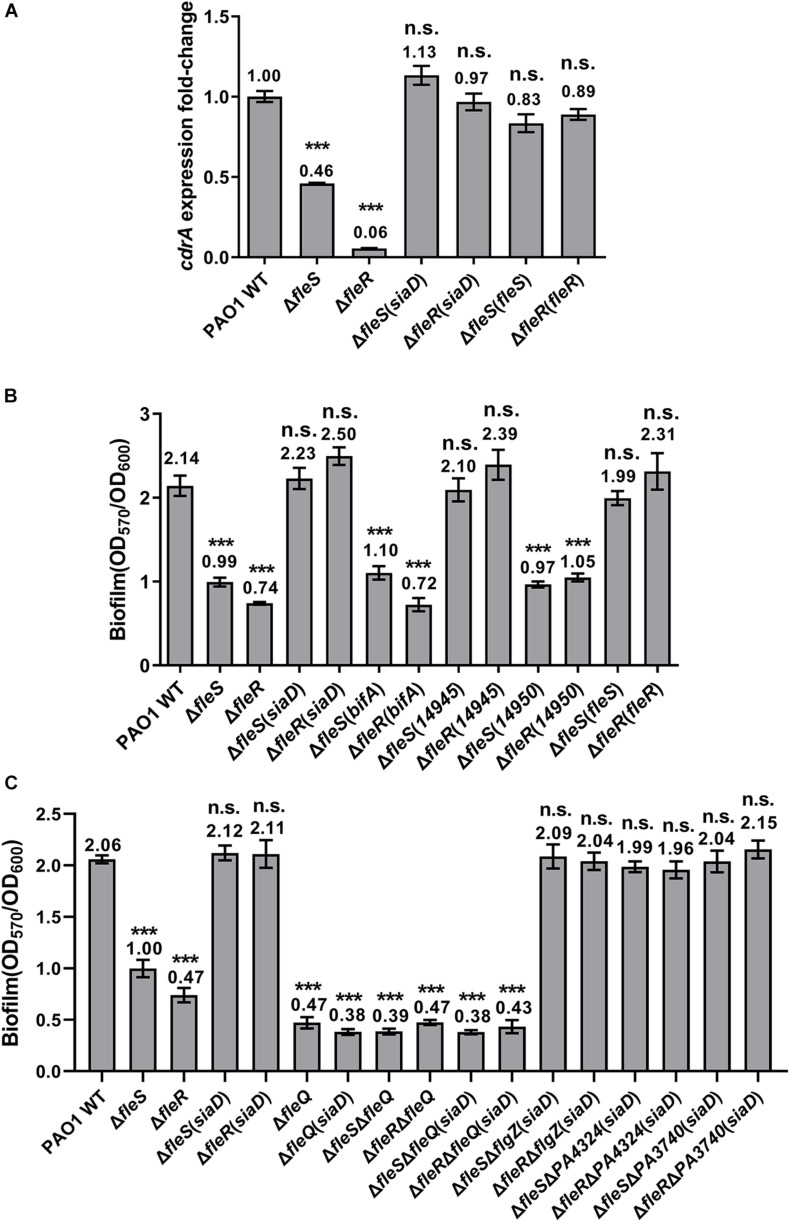
The regulation of FleS/FleR on biofilm formation is mediated by c-di-GMP. **(A)** Expression levels of *cdrA* were examined in the PAO1 wild type (WT), Δ*fleS* and Δ*fleR* mutants as well as the mutants with overexpression of a diguanylate cyclase gene *siaD*. The result is shown as relative *cdrA* expression compared to its wild-type level in PAO1. **(B)** Biofilm formation of the PAO1 wild type, Δ*fleS* and Δ*fleR* mutants as well as the mutants with overexpression of SiaD from PAO1 and W909_14945(14945) from *D. zeae* EC1. PDEs BifA from PAO1 and W909_14950(14950) from *D. zeae* EC1 serve as negative controls. **(C)** Examination of the effect of FleQ and other PilZ domain-containing proteins on biofilm formation in the presence of overexpressed SiaD. ***P < 0.001, n.s., no significance (Student’s *t*-test).

Since the key residues responsible for the signaling of the TCS FleS/FleR were genetically characterized, we then sought to investigate the mechanisms of how biofilm formation and swimming motility are regulated by FleS/FleR. Bacterial biofilm is a structured bacterial community which is mainly composed of polysaccharides, extracellular DNA, proteins and lipids (Flemming and Wingender, 2010). It has been demonstrated that biofilm formation in *P. aeruginosa* is associated with its intracellular c-di-GMP levels and a number of enzymes involved in the c-di-GMP metabolism have been identified that modulate the biofilm formation process (Ha and O’Toole, 2015). Cellular c-di-GMP is synthesized from two GTP molecules by diguanylate cyclases (DGC) and degraded by phosphodiesterases (PDE) (Hengge, 2009). Interestingly, our transcriptome data revealed that expression of diguanylate cyclase genes such as *siaD, sadC, gcbA, PA4929* were significantly repressed with log_2_fold changes ranging from −1.41 to −4.40 in the absence of FleR (Zhou et al., 2021), suggesting that reduced biofilm formation in the Δ*fleS* and Δ*fleR* mutants could be the consequence of decreased intracellular c-di-GMP contents. To test this hypothesis, we first measured the intracellular c-di-GMP contents using the expression level of *cdrA* as an indicator. CdrA is a member of the CdrAB two-partner secretion system and is positively regulated by the intracellular c-di-GMP molecules (Rybtke et al., 2012, 2015; Zhang et al., 2018). As shown in Figure 6A, deletion of *fleS* and *fleR* significantly repressed the expression of *cdrA*, indicating that intracellular c-di-GMP contents were indeed decreased in the absence of *fleS* and *fleR*.

To investigate whether decreased c-di-GMP contents led to the reduced biofilm formation as we observed in the Δ*fleS* and Δ*fleR* mutants, we attempted to increase the c-di-GMP levels and expected to observe elevated biofilm formation in these two mutants. We constructed a plasmid to overexpress a DGC gene *siaD* (Chen G. et al., 2020) and introduced it into the Δ*fleS* and Δ*fleR* mutants, respectively. We first measured the expression of *cdrA* which confirmed that the constructed *siaD*-overexpression plasmid was functional to increase the intracellular c-di-GMP content as indicated by the increased expression of *cdrA* (Figure 6A). Next, we found that biofilm formation in these two mutants was also increased to the level similar as that in the PAO1 wild type (Figure 6B). As a control, overexpression of *bifA*, a PDE gene participated in c-di-GMP degradation (Kuchma et al., 2007), was unable to restore the biofilm formation of these two mutants (Figure 6B). Owing that SiaD-mediated c-di-GMP metabolism has been demonstrated to interconnect with other signaling pathways in PAO1 to coordinate multiple cellular activities (Colley et al., 2016), we additionally overexpressed a DGC W909_14945 from *D. zeae* EC1 (Chen Y.F. et al., 2020) in the Δ*fleS* and Δ*fleR* mutants to ensure that overexpression of the DGC only elevates the intracellular c-di-GMP level. Consistent with the overexpression of SiaD, overexpression of W909_14945 restored biofilm formation in both Δ*fleS* and Δ*fleR* mutants to the wild-type level (Figure 6B). These results confirmed that the regulation of biofilm formation by FleS/FleR is mediated by intracellular c-di-GMP.

Given that c-di-GMP exerts its regulatory role through binding to specific effector proteins or RNAs (Boyd and O’Toole, 2012), we next moved to investigate which effector protein mediates the biofilm formation in this process. So far, various families of c-di-GMP effectors have been characterized which include proteins containing the PilZ domain, proteins containing degenerated GGDEF or EAL domains, transcriptional regulators, and mRNA riboswitches (Amikam and Galperin, 2006; Baraquet and Harwood, 2013). FleQ is the main effector for c-di-GMP signaling in *P. aeruginosa* which regulates exopolysaccharide (EPS) secretion to mediate the transition between planktonic and biofilm lifestyles (Baraquet et al., 2012). Thus, we first examined whether FleQ mediates the c-di-GMP signaling by deleting *fleQ* in PAO1 wild type, Δ*fleS* and Δ*fleR* mutants with or without overproduction of c-di-GMP in the cells. As shown in Figure 6C, deletion of *fleQ* abolished biofilm formation in all the strains in spite of the overproduction of intracellular c-di-GMP, indicating that c-di-GMP mediated biofilm formation requires FleQ. We further examined other PilZ domain-containing proteins such as FlgZ, PA4324 and PA3740 by deleting these genes in the Δ*fleS* and Δ*fleR* mutants with overproduction of c-di-GMP and monitored their biofilm formation. None of them showed reduced biofilm formation compared with their parental strains (Figure 6C), suggesting that these PilZ domain-containing proteins are not involved in biofilm formation in this case. Together, these findings demonstrated that the TCS FleS/FleR regulates biofilm formation by controlling the intracellular c-di-GMP level and the regulation is mediated by FleQ.

### FleR Regulates Flagellum Biosynthesis and Swimming Motility Independent of FleS

**FIGURE 7:**
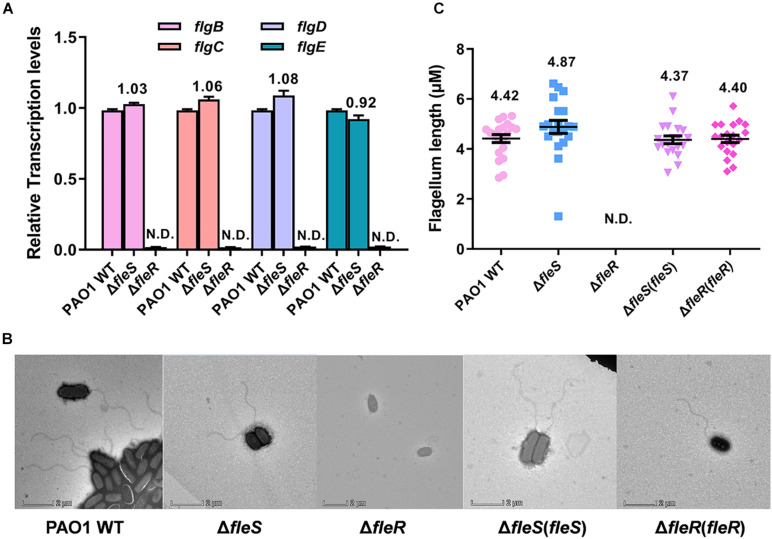
FleR regulates flagellum biosynthesis independent of FleS. **(A)** Expression levels of *flgB-E* were examined in the PAO1 wild type (WT), the Δ*fleS* and Δ*fleR* mutants. N.D., not detected. **(B)** Observation of flagellum in the PAO1 wild type, the Δ*fleS* and Δ*fleR* mutants as well as their corresponding *fleS* and *fleR* complemented strains by TEM. A representative image of each strain is shown. **(C)** The average flagellum length measured from the stains in panel **(B)**. Twenty cells were selected for the measurement from each strain.

Different from swarming motility which requires flagellum and biosurfactants, swimming is a unicellular behavior which depends on a functional polar flagellum and can be observed under microscopy (Rashid and Kornberg, 2000). Our study showed that swimming motility was slightly reduced by the deletion of *fleS* but completely abolished by the deletion of *fleR* (Figures 1C, 2C). This result led us to speculate that the regulation of FleS and FleR in swimming motility should be independent. It has been previously demonstrated that FleR can specifically recognize the promoters of *flgBCDE*, *flgFGHIJKL*, *fliC* which are responsible for the flagellum biosynthesis (Zhou et al., 2021), suggesting that FleR might influence bacterial swimming motility by controlling the synthesis of flagellum. To figure out if FleS/FleR regulates the synthesis of flagellum in PAO1, we firstly measured the expression of *flgBCDE* genes and found that the expression of *flg* genes was completely inhibited in the Δ*fleR* mutant while these genes still expressed ordinary in the Δ*fleS* mutant (Figure 7A). To further confirm this result, flagella of these strains were observed under the transmission electron microscope (TEM). Consistently, TEM results provided direct evidence that flagellum synthesis was completely inhibited in the mutant without FleR (Figure 7B). Thus, the absence of flagellum in the Δ*fleR* mutant explained its incapability of swimming. In contrast, regarding the Δ*fleS* mutant, it displayed intact flagellum which showed no difference in length as that of the PAO1 wild type (Figures 7B,C). These results together confirmed that FleR controls flagellum biosynthesis and swimming motility independent of its cognate sensor kinase FleS. Given that the AAA domain of FleR share 59% identity with that of FleQ and FleQ is a σ^54^-dependent master regulator involving flagellum biosynthesis (Supplementary Figure 3), it is highly possible that the AAA domain of FleR might enable the protein to execute regulation of flagellum biosynthesis and swimming motility without FleS. However, in turn, it remains unclear whether FleS regulates swimming through FleR owing to the integrity of flagellum in the Δ*fleS* mutant.

## Discussion

**FIGURE 8:**
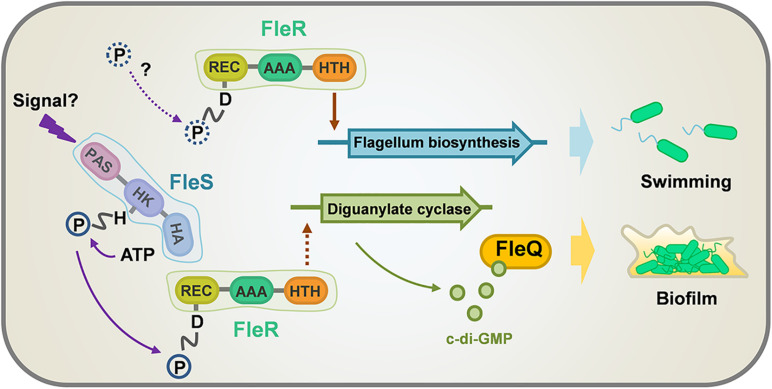
A model illustrating the regulation of swimming motility and biofilm formation by FleS and FleR in PAO1. Biofilm formation is regulated by both FleS and FleR in a c-di-GMP dependent manner which is mediated by another transcription factor FleQ. However, swimming motility is regulated discrepantly by FleS and FleR. FleR is demonstrated as a key regulator to independently control the biosynthesis of flagellum which is essential to support bacterial swimming, while FleS moderately regulates swimming motility through undetermined mechanisms. Dot lines indicate uncertain signaling pathways.

*P. aeruginosa* is a versatile opportunistic pathogen broadly distributed in different environments and a major cause of a variety of acute and chronic infections (Coggan and Wolfgang, 2012). TCS, consisting of a sensor histidine kinase (HK) and a cognate RR, is a predominant module to sense and respond to environmental changes (Stover et al., 2000). It plays important roles in regulating almost all aspects of bacterial physiology including its viability, growth, virulence, and antibiotic resistance. Thus, TCSs have ascended as convincing targets for the design of anti-microbial drugs (Tiwari et al., 2017). *P. aeruginosa* has a large genome which encodes at least 64 HKs and 73 RRs (Rodrigue et al., 2000). The abundant TCSs found in *P. aeruginosa* confer to this pathogen great adaptive ability to survive and thrive at various environmental and clinical niches. However, most of them are not characterized, which largely limits the anti-microbial exploitations. In the present study, we focused on a previously identified TCS FleS/FleR which is important in modulating multiple virulence-related traits in PAO1 (Zhou et al., 2021). We first investigated the signaling role of each domain and the key residues of FleS and FleR in controlling biofilm formation and swimming motility. Furthermore, we revealed that this TCS regulates biofilm formation in a c-di-GMP dependent manner, and this process is mediated by another transcription factor FleQ. Lastly, we showed that FleR is a key regulator functioning independent of FleS to control the biosynthesis of flagellum which is essential to support bacterial swimming (Figure 8).

Compared with canonical TCSs, FleS/FleR displays some unique features. Domain analysis showed that FleS consists of a PAS domain, a HisKA domain and a HATPase domain but lacks a transmembrane domain. PAS domain serves as a versatile sensor to detect chemical and physical stimuli (Taylor and Zhulin, 1999). HisKA domain is also known as the dimerization and histidine phosphotransfer (DHp) domain which contains a conserved histidine residue for phosphorylation (Stover et al., 2000). HATPase domain is a conserved catalytic and ATP-binding (CA) domain which transfers the phosphoryl group from ATP to the histidine residue (Jung and Altendorf, 1998). Sequence alignment and point mutagenesis indicated that the histidine at position 191 is the conserved residue for autophosphorylation. Given that FleS contains a PAS domain but without a transmembrane domain and all the three domains are demonstrated to be indispensable for FleS to control biofilm formation and swimming motility, FleS is implicated to be activated by some intracellular signals. We conducted sequence alignment between FleS-PAS and FixL-PAS which is a prototypic PAS domain binding heme for oxygen sensing (Delgado-Nixon et al., 2000; Key and Moffat, 2005) and identified a conversed amino acid residue L143 in FleS potentially involved in ligand binding (Supplementary Figure 4). It was recently reported that FleS mediates SDS-induced cell aggregation (Zhou et al., 2021), suggesting SDS is a possible ligand that can be recognized by FleS-PAS to initiate its signaling. However, the structural basis of ligand-binding and whether FleS-PAS perceives any other physiologically relevant signals requires further investigation.

Response regulator has a modular architecture which typically contains an N-terminal REC domain and a linked effector domain (West and Stock, 2001). The REC domain catalyzes the transfer of the phosphoryl group from the HK to its conserved aspartate residue and regulates the activity of the connected effector domain (Stover et al., 2000; West and Stock, 2001). FleR has three domains. In addition to the N-terminal REC domain, it also contains an AAA domain and a C-terminal HTH domain. Similarly, all the three domains are essential for the biofilm formation and swimming motility in PAO1. In the REC domain, D53 is speculated as the most potential residue for phosphorylation according to the results of point mutagenesis and sequence alignment. However, experiments also showed that aspartate residues at other positions such as D10, D11, D60 and D99 are also important for the FleR activity. D10 is supposed to bind cofactors because an octahedral coordination requiring D12 and D57 (conserved residues of D10 and D53 in FleR-REC, Supplementary Figure 5) was shown in the structure of Mg^2+^-bound CheY-REC (Stock et al., 1993; Bellsolell et al., 1994). Functions of other non-conserved aspartate residues remain uncertain. Furthermore, our study identified the key residues located in the AAA domain responsible for the FleR activity, i.e., K164, D229&E230, T208 of the Walker A motif, the Walker B motif and the σ^54^-binding region, with reference to the well-characterized key residues of PspE in *E. coli* and FleQ in *P. aeruginosa* (Schumacher et al., 2004; Joly et al., 2007; Baraquet and Harwood, 2013).

The regulation of FleS/FleR on biofilm formation is found meditated by the intracellular c-di-GMP levels. It seems FleR exhibits strong effect on the biosynthesis of c-di-GMP since deletion of *fleR* substantially reduced the expression of *cdrA*. We showed that overexpression of the diguanylate cyclases SiaD and W909_14945 in the mutants without FleS or FleR can fully recover their intracellular c-di-GMP contents and biofilm formation back to the wild-type levels. This process is specifically mediated by the regulator FleQ but not other PilZ domain-containing c-di-GMP effectors we examined. Residues R144, R185, N186, E330 and R334 are critical for c-di-GMP binding in FleQ (Matsuyama et al., 2016). Considering that R185 and N186 are located in the FleQ-AAA domain, we compared the sequences of FleQ and FleR to see if c-di-GMP also potentially binds to FleR. The result did not show any conserved key amino acid residues in FleR (Supplementary Figure 6), suggesting that FleR might not respond to c-di-GMP and only controls the c-di-GMP synthesis. It was previously reported that FleQ controls the transcription of FleS/FleR in a c-di-GMP dependent manner (Jimenez-Fernandez et al., 2016). Therefore, combined with our result, an interesting regulatory circuit of FleQ is suggested. On one hand, FleQ employs c-di-GMP to activates the transcription of FleS/FleR to maintain the constitutive biosynthesis of c-di-GMP. On the other hand, FleQ employs c-di-GMP to execute physiological regulation such as biofilm formation.

FleS/FleR has been reported to control flagellum biogenesis in *P. aeruginosa* (Dasgupta et al., 2003), which explains their importance in the motility. However, unlike their concordant effect on swarming motility (Kollaran et al., 2019), we found that FleS and FleR regulate swimming motility in different patterns. Since swimming depends on a functional polar flagellum (Rashid and Kornberg, 2000), we examined expression of genes responsible for flagellum biosynthesis and found their expression is merely controlled by FleR. TEM results provided direct evidence that flagellum assembly is only abolished in the Δ*fleR* mutant but not the Δ*fleS* mutant. Combined with a recent study which showed that non-phosphorylated FleR can specifically bind to the promoters of flagellum biosynthetic operons and auto-regulate its own expression without FleS (Zhou et al., 2021), we speculated that regulation of flagellum biosynthesis by FleR might be unrelated to its phosphorylation status or it could be phosphorylated by other histidine kinase in addition to FleS. Moreover, it is reminded that AAA domains are involved in a variety of cellular activities owing to their ATPase activity (Seraphim and Houry, 2020). Hence, there is another possibility that the FleR-AAA domain supports chemical energy for flagellum biosynthesis. For FleS, it is still not known how to regulate the swimming motility owing to their irrelevance with the flagellum biosynthesis.

*P. aeruginosa* infection is a dynamic adaptive process which starts from acute infection to a finally host-adapted chronic infection (Sousa and Pereira, 2014). During this transition, *P. aeruginosa* undergoes numerous physiological changes which are coordinated by complicated regulatory systems including TCS. In fact, motility and biofilm formation represent two important pathogenic traits of *P. aeruginosa* acute and chronic infections, respectively (Balasubramanian et al., 2013). Interestingly, our study found that both traits are positively regulated by the TCS FleS/FleR, indicating that FleS/FleR is essential for *P. aeruginosa* to establish both acute and chronic infections. Whether FleR dominates without FleS at the stage of acute infection to control flagellum biosynthesis and gradually shifts to facilitate biofilm formation in a FleS and c-di-GMP dependent manner at the stage of chronic infection warrants further investigations. Nonetheless, FleS/FleR could be a promising target to be exploited for infection control.

## Data Availability Statement

The original contributions presented in the study are included in the article/Supplementary Material, further inquiries can be directed to the corresponding authors.

## Author Contributions

TZ, ZX, and L-hZ designed the experiments. TZ, JH, and ZL conducted the experiments. TZ and ZX performed the data analysis. TZ, ZX, and L-hZ wrote the manuscript. All authors contributed to the article and approved the submitted version.

## Conflict of Interest

The authors declare that the research was conducted in the absence of any commercial or financial relationships that could be construed as a potential conflict of interest.

## Supplementary Material

Supplementary Figure 1 Stable expression of the FleS/FleR variants constructed in this study. Variants of FleS and FleR used in this study are His-tagged and examined by western blot.

Supplementary Figure 2 Schematic diagram showing the aspartate residues in the FleR-REC domain. The positions of each aspartate residue are shown.

Supplementary Figure 3 Alignment of the AAA domains from FleR and FleQ. Residues shown with 100% identity are highlighted in red.

Supplementary Figure 4 Alanine substitution of leucine at the position of 143 in the FleS-PAS domain inactivates the signaling activity of FleS.

Supplementary Figure 5 Alignment of the REC sequences from CheY and FleR. D12 and D57 in CheY with corresponding to the D10 and D53 residues in FleR are marked with red boxes.

Supplementary Figure 6 Sequence alignment of FleQ and FleR. Residues R144, R185, N186, E330, and R334 which are critical for c-di-GMP binding in FleQ are highlighted in red boxes.

Supplementary Table 1 Bacterial strains and plasmids used in this study.

Supplementary Table 2 PCR primers used in this study.
