# Supplementary material for: Molecular Mechanisms Underlying the Regulation of Biofilm Formation and Swimming Motility by FleS/FleR in Pseudomonas aeruginosa
Source: Front Microbiol. 2021 Jul 21;12:707711. doi: 10.3389/fmicb.2021.707711 (PMC8335546; doi:10.3389/fmicb.2021.707711)
Supplement: Supplementary Figure 1 — Stable expression of the FleS/FleR variants constructed in this study. Variants of FleS and FleR used in this study are His-tagged and examined by western blot. [file Data_Sheet_1.DOCX]

**Supplementary Data**

**Table S1**. Bacterial strains and plasmids used in this study.

| Strain or plasmid | Relevant genotype or phenotype | Source or reference |
| --- | --- | --- |
| ***P.aeruginose* strains** |  |  |
| PAO1 | Wild type strain | Lab collection |
| ∆*fleS* | *fleS* deletion mutant of PAO1 with 1208-nt internal coding region deleted | This study |
| ∆*fleR* | *fleR* deletion mutant of PAO1 with 1422-nt internal coding region deleted | This study |
| ∆*fleS*∆*fleR* | ∆*fleS* ∆*fleR* double-deletion mutant | This study |
| ∆*fleS*(*fleS*) | Mutant ∆*fleS* harboring the expression construct pBBR1-MCS5-*fleS* | This study |
| ∆*fleR*(*fleR*) | Mutant ∆*fleR* harboring the expression construct pBBR1-MCS5-*fleS* | This study |
| ∆*fleS*∆*fleR*(*fleS*) | Double-deletion mutant ∆*fleS*∆*fleR* harboring the expression construct pBBR1-MCS5-*fleS* | This study |
| ∆*fleS*∆*fleR*(*fleR*) | Double-deletion mutant ∆*fleS*∆*fleR* harboring the expression construct pBBR1-MCS5-*fleR* | This study |
| ∆*REC* | REC domain of *fleR* deletion mutant | This study |
| ∆*AAA* | AAA domain of *fleR* deletion mutant | This study |
| ∆*HTH* | HTH domain of *fleR* deletion mutant | This study |
| ∆*REC*(*REC*) | ∆*REC* harboring the expression construct  pBBR1-MCS5-REC domain of *fleR* | This study |
| ∆*AAA*(*AAA*) | ∆*AAA* harboring the expression construct pBBR1-MCS5-AAA domain of *fleR* | This study |
| ∆*HTH*(*HTH*) | ∆*HTH* harboring the expression construct pBBR1-MCS5-HTH domain of *fleR* | This study |
| ∆*REC*(*fleR*) | ∆*REC* harboring the expression construct pBBR1-MCS5-*fleR* | This study |
| ∆*AAA*(*fleR*) | ∆*AAA* harboring the expression construct pBBR1-MCS5-*fleR* | This study |
| ∆*HTH*(*fleR*) | ∆*HTH* harboring the expression construct pBBR1-MCS5- *fleR* | This study |
| ∆*fleS*(*fleS*∆*PAS*) | Mutant ∆*fleS* harboring the expression construct pBBR1-MCS5-*fleS* of absence PAS domain | This study |
| ∆*fleS*(*fleS*∆*HisKA*) | Mutant ∆*fleS* harboring the expression construct pBBR1-MCS5-*fleS* of absence HisKA domain | This study |
| ∆*fleS*(*fleS*∆*HATPase*) | Mutant ∆*fleS* harboring the expression construct pBBR1-MCS5-*fleS* of absence HATPase domain | This study |
| ∆*fleR*(*fleR*∆*REC*) | Mutant ∆*fleR* harboring the expression construct pBBR1-MCS5-*fleR* of absence REC domain | This study |
| ∆*fleR*(*fleR*∆*AAA*) | Mutant ∆*fleR* harboring the expression construct pBBR1-MCS5-*fleR* of absence AAA domain | This study |
| ∆*fleR*(*fleR*∆*HTH*) | Mutant ∆*fleR* harboring the expression construct pBBR1-MCS5-*fleR* of absence HTH domain | This study |
| ∆*fleS*(*fleS^H191A^*) | Mutant ∆*fleS* harboring the expression construct pBBR1-MCS5-*fleS^H191A^* | This study |
| ∆*fleS*(*fleS^I135A^*) | Mutant ∆*fleS* harboring the expression construct pBBR1-MCS5-*fleS^I135A^* | This study |
| ∆*fleS*(*fleS^L143A^*) | Mutant ∆*fleS* harboring the expression construct pBBR1-MCS5-*fleS^L143A^* | This study |
| ∆*fleS*(*fleS^I135AL143A^*) | Mutant ∆*fleS* harboring the expression constructpBBR1-MCS5-*fleS^I135AL143A^* | This study |
| ∆*fleR*(*fleR^D10A^*) | Mutant ∆*fleR* harboring the expression construct pBBR1-MCS5-*fleR^D10A^* | This study |
| ∆*fleR*(*fleR^D53A^*) | Mutant ∆*fleR* harboring the expression construct pBBR1-MCS5-*fleR^D53A^* | This study |
| ∆*fleR*(*fleR^D99A^*) | Mutant ∆*fleR* harboring the expression construct pBBR1-MCS5-*fleR^D99A^* | This study |
| ∆*fleR*(*fleR^D11A^*) | Mutant ∆*fleR* harboring the expression construct pBBR1-MCS5-*fleR^D11A^* | This study |
| ∆*fleR*(*fleR^D20A^*) | Mutant ∆*fleR* harboring the expression construct pBBR1-MCS5-*fleR^D20A^* | This study |
| ∆*fleR*(*fleR^D33A^*) | Mutant ∆*fleR* harboring the expression construct pBBR1-MCS5-*fleR^D33A^* | This study |
| ∆*fleR*(*fleR^D60A^*) | Mutant ∆*fleR* harboring the expression construct pBBR1-MCS5-*fleR^D60A^* | This study |
| ∆*fleR*(*fleR^D87A^*) | Mutant ∆*fleR* harboring the expression construct pBBR1-MCS5-*fleR^D87A^* | This study |
| ∆*fleR*(*fleR^D112A^*) | Mutant ∆*fleR* harboring the expression construct pBBR1-MCS5-*fleR^D112A^* | This study |
| ∆*fleR*(*fleR^K180A^*) | Mutant ∆*fleR* harboring the expression construct pBBR1-MCS5-*fleR^K180A^* | This study |
| ∆*fleR*(*fleR^T208A^*) | Mutant ∆*fleR* harboring the expression construct pBBR1-MCS5-*fleR^T208A^* | This study |
| ∆*fleR*(*fleR^D229AE230A^*) | Mutant ∆*fleR* harboring the expression construct pBBR1-MCS5-*fleR^D229AE230A^* | This study |
| ∆*fleQ* | *fleQ* deletion mutant of PAO1 which the gene *fleQ* was in frame deleted | This study |
| ∆*fleS*∆*fleQ* | *fleS* and *fleQ* double deletion mutant which *fleQ* was deleted in ∆*fleS* background | This study |
| ∆*fleR*∆*fleQ* | *fleR* and *fleQ* double deletion mutant which *fleQ* was deleted in ∆*fleR* background | This study |
| ***E. coli* strains** |  |  |
| DH5α | *spuE44 ∆lacU169(φ80lacZ∆M15) hsdR17λpir recA1 endA1 gyrA96 thi-1 relA1* | Lab collection |
| BL21(DE3) | F^-^*ompT* *hsdS* (r_B_^-^m_B_^-^) *dcm*^+^ Tet^r^ *gal* (DE3) *endA* | Lab collection |
| pRK2013 | Tra^+^, Mob^-^, ColE1-replicon, Kan^r^, Spe^r^ | Lab collection |
| **Plasmids** |  |  |
| pBBR1-MCS5 | Broad host-range cloning vector; Gm^r^ | Lab collection |
| pBBR1-MCS5-*fleS* | pBBR1-MCS5 containing *fleS* under control of P_lac_ | This study |
| pBBR1-MCS5-*fleR* | pBBR1-MCS5 containing *fleR* under control of P_lac_ | This study |
| pK18mobsacB | Broad-host-range gene replacement vector, sacB,Gm^r^ | Lab collection |
| pK18-*fleS* | pK18 containing *fleS* flanking regions for generation of *fleS* in-frame deletion | This study |
| pK18-*fleR* | pK18 containing *fleR* flanking regions for generation of *fleR* in-frame deletion | This study |
| pBBR1-MCS5-Gm-*lacZ* | lacZ transcriptional fusion vector, Gm^r^ | Lab collection |
| pBBR1-MCS5-*fleS* | Single-copy Tn7 insertion plasmid containing *fleS* | This study |
| pBBR1-MCS5-*fleR* | Single-copy Tn7 insertion plasmid containing *fleR* | This study |
| pBBR1-MCS5-*fleS^H191A^* | pBBR1-MCS5 containing *fleS* derivative with H191A substitution | This study |
| pBBR1-MCS5-*fleS^I135A^* | pBBR1-MCS5 containing *fleS* derivative with I135A substitution | This study |
| pBBR1-MCS5-*fleS^L143A^* | pBBR1-MCS5 containing *fleS* derivative with L143A substitution | This study |
| pBBR1-MCS5-*fleS^I135AL143A^* | pBBR1-MCS5 containing *fleS* derivative with I135A and L143A substitution | This study |
| pBBR1-MCS5-*fleR^D10A^* | pBBR1-MCS5 containing *fleR* derivative with D10A substitution | This study |
| pBBR1-MCS5-*fleR^D53A^* | pBBR1-MCS5 containing *fleR* derivative with D53A substitution | This study |
| pBBR1-MCS5-*fleR^D99A^* | pBBR1-MCS5 containing *fleR* derivative with D99A substitution | This study |
| pBBR1-MCS5-*fleR^D11A^* | pBBR1-MCS5 containing *fleR* derivative with D11A substitution | This study |
| pBBR1-MCS5-*fleR^D20A^* | pBBR1-MCS5 containing *fleR* derivative with D20A substitution | This study |
| pBBR1-MCS5-*fleR^D33A^* | pBBR1-MCS5 containing *fleR* derivative with D33A substitution | This study |
| pBBR1-MCS5-*fleR^D60A^* | pBBR1-MCS5 containing *fleR* derivative with D60A substitution | This study |
| pBBR1-MCS5-*fleR^D87A^* | pBBR1-MCS5 containing *fleR* derivative with D87A substitution | This study |
| pBBR1-MCS5-*fleR^D112A^* | pBBR1-MCS5 containing *fleR* derivative with D112A substitution | This study |
| pBBR1-MCS5-*fleR^K180A^* | pBBR1-MCS5 containing *fleR* derivative with K180A substitution | This study |
| pBBR1-MCS5-*fleR^T208A^* | pBBR1-MCS5 containing *fleR* derivative with T208A substitution | This study |
| pBBR1-MCS5-*fleR^D229AE230A^* | pBBR1-MCS5 containing *fleR* derivative with D229A and E230A substitution | This study |

Gm^r^, gentamicin resistance; Kan^r^, kanamycin resistance; Amp^r^, ampicillin resistance

**Table S2.** PCR primers used in this study.

| Primers | Sequence (5’-3’) | Application |
| --- | --- | --- |
| *fleS*-Up-F | gagctcggtacccggggatccGGAGCGCCTGGCGATCAT | For amplification of the 5’-region of *fleS* |
| *fleS*-Up-R | aatcgcagaaagGGCGTTGAGGGCTGGTTG |  |
| *fleS*-Dn-F | tcaacgccCTTTCTGCGATTCAGGAGTAACC | For amplification of the 3’-region of *fleS* |
| *fleS*-Dn-R | acgacggccagtgccaagcttCGCCGGAGATCAGCACGG |  |
| *fleR*-Up-F | gagctcggtacccggggatccGCTGGTGTTCGCCCGCGG | For amplification of the 5’-region of *fleR* |
| *fleR*-Up-R | agcacGGGGTTACTCCTGAATCGCAG |  |
| *fleR*-Dn-F | ttcaggagtaaccccGTGCTCGCCATGTTCCCC | For amplification of the 3’-region of *fleR* |
| *fleR*-Dn-R | acgacggccagtgccaagcttACGCTGGCCTTCTGGCTG |  |
| *fleR*-De-REC-1 | gagctcggtacccggggatccGCTGGTGTTCGCCCGCGG | For amplification of the 5’-region REC domain of *fleR* |
| *fleR*-De-REC-2 | TGCCATGGGGTTACTCCTGA |  |
| *fleR*-De-REC-3 | tcaggagtaaccccatggcaGCGCGCCATGCGCTGGGC | For amplification of the 3’-region REC domain of *fleR* |
| *fleR*-De-REC-4 | acgacggccagtgccaagcttACGCTGGCCTTCTGGCTG |  |
| *fleR*-De-AAA-1 | gagctcggtacccggggatccGCTGGTGTTCGCCCGCGG | For amplification of the 5’-region AAA domain of *fleR* |
| *fleR*-De-AAA-2 | ccagcgggaaGCGCGCGACCCGCGCGGC |  |
| *fleR*-De-AAA-3 | ggtcgcgcgcTTCCCGCTGGCCTGGCGG | For amplification of the 3’-region AAA domain of *fleR* |
| *fleR*-De-AAA-4 | acgacggccagtgccaagcttACGCTGGCCTTCTGGCTG |  |
| *fleR*-De-HTH-1 | gagctcggtacccggggatccGCTGGTGTTCGCCCGCGG | For amplification of the 5’-region HTH domain of *fleR* |
| *fleR*-De-HTH-2 | tccatccccgcgtcgcgGTCGCCCAGCGCGCCGCT |  |
| *fleR*-De-HTH-3 | gacCGCGACGCGGGGATGGAC | For amplification of the 3’-region HTH domain of *fleR* |
| *fleR*-De-HTH-4 | acgacggccagtgccaagcttACGCTGGCCTTCTGGCTG |  |
| *fleS*-FC-F | gtcgacggtatcgataagcttGGGGTGATCGGGGTCGGC | For construction of pBBR1-MCS5-*fleS* |
| *fleS*-FC-R | cgctctagaactagtggatccCGCGTAGTGCGCGGTCGT |  |
| *fleR*-FC-F | gtcgacggtatcgataagcttTGCGGGCCCGAACTGCGC | For construction of pBBR1-MCS5-*fleR* |
| *fleR*-FC-R | cgctctagaactagtggatccGCGGACGCAAAAGGCCCG |  |
| *sadC*-PC-F | gtcgacggtatcgataagcttCGGGTCGGCCAGGATCGA | For construction of pBBR1-MCS5-*sadC* |
| *sadC*-PC-R | cgctctagaactagtggatccAGGTTGCTGCCGGCGGCG |  |
| *bifA*-PC-F | gtcgacggtatcgataagcttCGACGTCTGGGAACACGC | For construction of pBBR1-MCS5-*bifA* |
| *bifA*-PC-R | cgctctagaactagtggatccCTGGGCAGCGCGCTATTG |  |
| *siaD*-PC-F | gtcgacggtatcgataagcttCAAGGCCATGATGGACATCC | For construction of pBBR1-MCS5-*siaD* |
| *siaD*-PC-R | cgctctagaactagtggatccCTCAGCCGAGGCCAGGTG |  |
| *fleS*-De-PAS-1 | gtcgacggtatcgataagcttATGCAACCAGCCCTCAACG | For construction the 5’-region of pBBR-*fleS*(∆PAS) |
| *fleS*-De-PAS-2 | gtcCGCCAGGCGCTCCTTTTC |  |
| *fleS*-De-PAS-3 | aaaaggagcgcctggcgGACGGACGTCGCCTGTCC | For construction the 3’-region of pBBR-*fleS*(∆PAS) |
| *fleS*-De-PAS-4 | cgctctagaactagtggatccTTACTCCTGAATCGCAGAAA |  |
| *fleS*-De-HisKA-1 | gtcgacggtatcgataagcttATGCAACCAGCCCTCAACG | For construction the 5’-region of pBBR-*fleS*(∆HisKA) |
| *fleS*-De-HisKA-2 | gtcggtcagCGACAGGCGCTCGTGGCG |  |
| *fleS*-De-HisKA-3 | agcgcctgtcgCTGACCGACCGGGTGGCG | For construction the 3’-region of pBBR-*fleS*(∆HisKA) |
| *fleS*-De-HisKA-4 | cgctctagaactagtggatccTTACTCCTGAATCGCAGAAA |  |
| *fleS*-De-HATPase-F | gtcgacggtatcgataagcttATGCAACCAGCCCTCAACG | For construction the of pBBR-*fleS*(∆HATPase) |
| *fleS*-De-HATPase-R | cgctctagaactagtggatccTTACTCCTGAATCGCAGAAAGAGGCGCCGCCAGCAGCTCGCCGCCGCG |  |
| *fleR*-De-REC-F | gtcgacggtatcgataagcttATGGCAGCGCGCCATGCGCTGGGC | For construction the of pBBR-*fleR*(∆REC) |
| *fleR*-De-REC-R | cgctctagaactagtggatccTCAGATGGCGTAGAGATAGGCC |  |
| *fleR*-De-AAA-UF | gtcgacggtatcgataagcttATGGCAGCCAAAGTCCTGC | For construction the 5’-region of pBBR-*fleR*(∆AAA) |
| *fleR*-De-AAA-UR | ccagcgggaaGCGCGCGACCCGCGCGGC |  |
| *fleR*-De-AAA-DF | ggtcgcgcgcTTCCCGCTGGCCTGGCGG | For construction the 3’-region of pBBR-*fleR*(∆AAA) |
| *fleR*-De-AAA-DR | cgctctagaactagtggatccTCAGATGGCGTAGAGATAGGCC |  |
| *fleR*-De-HTH-F | gtcgacggtatcgataagcttATGGCAGCCAAAGTCCTGC | For construction the of pBBR-*fleR*(∆HTH) |
| *fleR*-De-HTH-R | cgctctagaactagtggatccTCAGATGGCGTAGAGATAGGCCTCCACGTCCATCCCCGCGTCGCGGTCGCCCAGCGCGCCGCT |  |
| *fleR^D10A^*-F | gtcgacggtatcgataagcttATGGCAGCCAAAGTCCTGCTGGTCGAAGCCGACCGCGCACTACGCGAA | For construction of pBBR-*fleR*(D10A) |
| *fleR^D10A^*-R | cgctctagaactagtggatccTCAGATGGCGTAGAGATAGGCC |  |
| *fleR^D11A^*-F | gtcgacggtatcgataagcttATGGCAGCCAAAGTCCTGCTGGTCGAAGACGCCCGCGCACTACGCGAAGCC | For construction of pBBR-*fleR*(D11A) |
| *fleR^D11A^*-R | cgctctagaactagtggatccTCAGATGGCGTAGAGATAGGCC |  |
| *fleR^D20A^*-2 | tGGCGCTGAGGGCTTCGCGTAGT | For construction the 5’-region of pBBR-*fleR*(D20A) with *fleR*-PC-F primer |
| *fleR^D20A^*-3 | actacgcgaagccctcagcGCCACCCTGCTGCTGGGCGGT | For construction the 3’-region of pBBR-*fleR*(D20A) with *fleR*-PC-R primer |
| *fleR^D33A^*-2 | aGGCCACGGCGACGAACTCGTG | For construction the 5’-region of pBBR-*fleR*(D33A) with *fleR*-PC-F primer |
| *fleR^D33A^*-3 | tcacgagttcgtcgccgtgGCCTCGGCGGAGGCGGCGCTG | For construction the 3’-region of pBBR-*fleR*(D33A) with *fleR*-PC-R primer |
| *fleR^D53A^*-2 | catgttcacGCCGCTGATCACCAGGCTGAAGG | For construction the 5’-region of pBBR-*fleR*(D53A) with *fleR*-PC-F primer |
| *fleR^D53A^*-3 | tggtgatcagcGGCGTGAACATGCCGGGCATG | For construction the 3’-region of pBBR-*fleR*(D53A) with *fleR*-PC-R primer |
| *fleR^D60A^*-2 | agcaactggtgtccGGCCATGCCCGGCATGTTCAC | For construction the 5’-region of pBBR-*fleR*(D60A) with *fleR*-PC-F primer |
| *fleR^D60A^*-3 | ggcatgGCCGGACACCAGTTGCTCGGCC | For construction the 3’-region of pBBR-*fleR*(D60A) with *fleR*-PC-R primer |
| *fleR^D87A^*-2 | atcgcctcgacggcgcgGGCGACCGCGCCGTAGGCGGT | For construction the 5’-region of pBBR-*fleR*(D87A) with *fleR*-PC-F primer |
| *fleR^D87A^*-3 | gtcGCCCGCGCCGTCGAGGCGATG | For construction the 3’-region of pBBR-*fleR*(D87A) with *fleR*-PC-R primer |
| *fleR^D99A^*-2 | tcgaacggcttgaccaggtaGGCGGCGGCGCCCTGGCGCAT | For construction the 5’-region of pBBR-*fleR*(D99A) with *fleR*-PC-F primer |
| *fleR^D99A^*-3 | GCCTACCTGGTCAAGCCGTTCGA | For construction the 3’-region of pBBR-*fleR*(D99A) with *fleR*-PC-R primer |
| *fleR^D112A^*-2 | atggcgcgccaccagGGCGAGCAGCGCCCGCGCCTC | For construction the 5’-region of pBBR-*fleR*(D112A) with *fleR*-PC-F primer |
| *fleR^D112A^*-3 | tgctcGCCCTGGTGGCGCGCCATGCG | For construction the 3’-region of pBBR-*fleR*(D112A) with *fleR*-PC-R primer |
| *fleR^K164A^*-2 | agttggccaggacttcTGCGCCGGTCCCGGACTCGCC | For construction the 5’-region of pBBR-*fleR*(K164A) with *fleR*-PC-F primer |
| *fleR^K164A^*-3 | cGCAGAAGTCCTGGCCAACTATATCCA | For construction the 3’-region of pBBR-*fleR*((K164A) with *fleR*-PC-R primer |
| *fleR^T208A^*-2 | ggcgccTGCGAAGGAACCTTTCTCGTGGCC | For construction the 5’-region of pBBR-*fleR*(T208A) with *fleR*-PC-F primer |
| *fleR^T208A^*-3 | aaggttccttcGCAGGCGCCATTGCCGCCCAG | For construction the 3’-region of pBBR-*fleR*(T208A) with *fleR*-PC-R primer |
| *fleR^D229AE230A^*-2 | tttccgatatTGCTGCGAGAAGAATGGTGCCGCCG | For construction the 5’-region of pBBR-*fleR*(D229AE230A) with *fleR*-PC-F primer |
| *fleR*^D229AE230A^-3 | tctcGCAGCAATATCGGAAATGCCCCTCG | For construction the 3’-region of pBBR-*fleR*(D229AE230A) with *fleR*-PC-R primer |
| *fleS^I135A^*-2 | cagggaCGCCTCGTGACCGTCATCCTCGC | For construction the 5’-region of pBBR-*fleR*(I135A) with *fleS*-PC-F |
| *fleS^I135A^*-3 | atgacggtcacgaggcgTCCCTGCGCGACGGACGT | For construction the 3’-region of pBBR-*fleR*(I135A) with *fleS*-PC-R |
| *fleS^L143^*^A^-2 | tggcgatggaCGCGCGACGTCCGTCGCGCAG | For construction the 5’-region of pBBR-*fleR*(L143A) with *fleS*-PC-F |
| *fleS^L143A^*-3 | cggacgtcgcgcgTCCATCGCCACCCGCTCG | For construction the 5’-region of pBBR-*fleR*(L143A) with *fleS*-PC-R |
| *fleS^H191A^*-2 | aacggcgtgcggatctgcgcGGCCAGCGAGGCGACCAT | For construction the 5’-region of pBBR-*fleR*(H191A) with *fleS*-PC-F |
| *fleS^H191A^*-3 | GCGCAGATCCGCACGCCG | For construction the 5’-region of pBBR-*fleR*(H191A) with *fleS*-PC-R |
| *fleR*-Up-F | gagctcggtacccggggatccATCGGTGAGCTGGATCAGGTC | For amplification of the 5’-region of *fleQ* |
| *fleR*-Up-R | atccgaTTCGCGCCACATTTTGATC |  |
| *fleR*-Dn-F | aaatgtggcgcgaaTCGGATGATTGACAGGTCGTT | For amplification of the 3’-region of *fleQ* |
| *fleR*-Dn-R | acgacggccagtgccaagcttCCTCGCGCGGAGCGAAGC |  |
| q-*flgB*-F | GAGCACTCGGTATCCACCAG | For qRT-PCR analysis  For qRT-PCR analysis |
| q-*flgB*-R | GATATGCCGCTCGTTGGTG |  |
| q-*flgC*-F | CCTCGCCAGTGTCTTCAACA |  |
| q-*flgC*-R | CTGCTGGAACATGGTGGAGA |  |
| q-*flgD*-F | GTCGCTGAACAAGAGCATGG |  |
| q-*flgD*-R | TTGACCCATACGTTGCTGCT |  |
| q-*flgF*-F | GTGCTCATGCCAACAACCTG |  |
| q-*flgF*-R | GCCATGGCTGAAATCGGT |  |
| q-*cdrA*-F | CCAGTTCAACCCCAACGAGA |  |
| q-*cdrA*-R | GTCGAAGCCCTTCCAGTTGA |  |
| *rplU*-F | GCAGCACAAAGTCACCGAAG | Internal control for qRT-PCR analysis |
| *rplU*-R | CCGATTTTCACGTCTTCGCC |  |
| *W909_-_14945*-FC-F | GTCGACGGTATCGATAAGCTTAAATATTAGCCAGGCTTATGT | For construction of pBBR1-MCS5-*W909_-_14945* |
| *W909_-_14945*-FC-R | CGCTCTAGAACTAGTGGATCCCTAGTGGTGGTGGTGGTGGTGCTGGTGTGTATTCAGATCA |  |
| *W909_-_14950*-FC-F | GTCGACGGTATCGATAAGCTTTACACAGCGTGGTTATGGTATA | For construction of pBBR1-MCS5-*W909_-_14950* |
| *W909_-_14950*-FC-R | CGCTCTAGAACTAGTGGATCCTTAGTGGTGGTGGTGGTGGTGCAGCTCTGAACGGAGATCTT |  |


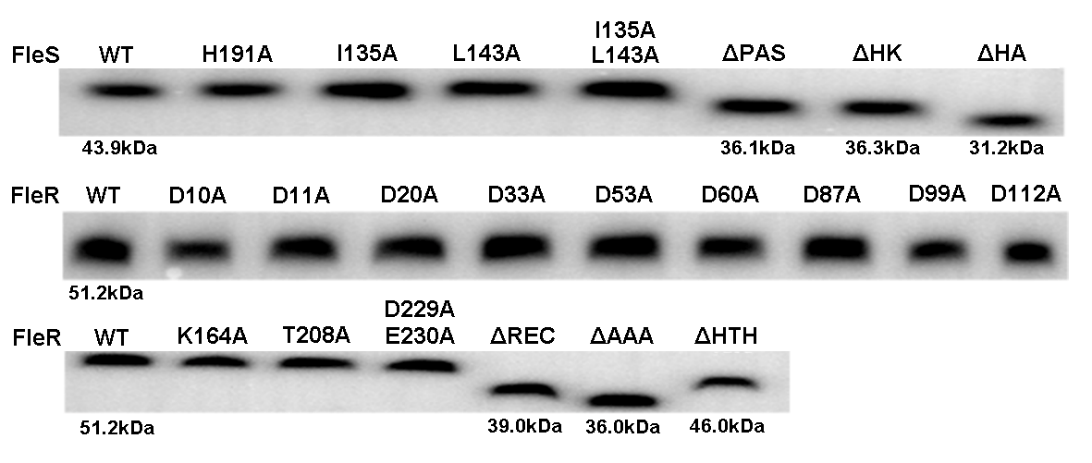


**Figure S1. Stable expression of FleS/FleR variants constructed in this study.** Variants of FleS and FleR used in this study are His-tagged and examined by western blot.

**Figure S2. Schematic diagram showing the aspartate residues in the FleR-REC domain**. The positions of each aspartate residue are shown.

**Figure S3. Alignment of the AAA domains from FleR and FleQ**. Residues shown with 100% identity are highlighted in red.

**Figure S4. Alanine substitution of leucine at the position of 143 in the FleS-PAS domain inactivates the signaling activity of FleS.**

**Figure S5. Alignment of the REC sequences from CheY and FleR**. D12 and D57 in CheY with corresponding to the D10 and D53 residues in FleR are marked with red boxes.

**Figure S6. Sequence alignment of FleQ and FleR**. Residues R144, R185, N186, E330 and R334 which are critical for c-di-GMP binding in FleQ are highlighted in red boxes.
